# Associations Between Hair Heavy Metal Concentrations and Frailty in Mexican Adults: a Cross-Sectional Study

**DOI:** 10.21203/rs.3.rs-10010697/v1

**Published:** 2026-06-15

**Authors:** Mario Ulises Pérez-Zepeda, Karla Julieta Esquivel-García, María José Pérez-Guzmán, Natalia Sánchez-Garrido, Miguel Germán Borda

**Keywords:** frailty, heavy metals, social vulnerability, Older adults

## Abstract

**Background:**

Exposure to heavy metals is increasingly recognized as a potential risk factors for health conditions and its accumulation across life has become of interes in older adults. Older adults may be susceptible because of accumulated exposure, reduced clearance, and greater vulnerability. Whether heavy metals contribute to frailty in community-dwelling adults, and whether their effect is modified by social vulnerability or sex, remains unclear.

**Methods.:**

Cross-sectional analysis of 2018 wave of the Mexican Health and Aging Study (MHAS). Hair samples from 2,671 participants aged 18 to 99 years were analyzed (EPA method 6020B). Frailty was operationalized with a 60-item frailty index (FI). Associations between each log-metal and FI were estimated with crude and covariate-adjusted linear (OLS) regression with heteroscedasticity-consistent (HC3) standard errors, and with fractional-logit regression. False-discovery-rate correction was applied. Effect modification by SVI and by sex was assessed.

**Results.:**

2,590 participants had a valid FI. In crude analyses, most metals were inversely associated with the FI. After adjustment, only cobalt retained inversely associated, which did not survive FDR correction. No metals were significant in multivariable models. Inverse association of vanadium and copper with the FI was confined to participants with higher social vulnerability; at low SVI these slopes were null or positive.

**Conclusions.:**

In this sample, individual hair concentrations of heavy metals were not robustly associated with frailty after adjustment. The results suggest that the relationship between trace metals and frailty may be small and highly context-dependent.

## Introduction

Population aging and environmental pollution are two of the most pressing public-health challenges of the twenty-first century, raising numerous questions on how these pollutants have the potential to impact human health [1]. Among those environmental factors, air pollution is characterized by the presence of heavy metals, several of which are physiologically necessary in trace amounts but become toxic above certain thresholds [2]. These risk factors have shown to be associated with negative health outcomes in particular in those populations considered to be vulnerable as a result of their life stage (e.g., children, pregnant women, older adults) [3].

Exposure to heavy metals occurs through inhalation of airborne particles, ingestion of contaminated water or food, and through the food chain [3]. Accumulation in tissues over decades results in multiple toxicity mechanisms: lead and vanadium interfere with enzyme activity, causing organ-level alterations [4,5]; arsenic, mercury, manganese and copper at elevated concentrations induce neurotoxicity and have been linked to cognitive decline and neurodegenerative disorders such as Alzheimer's and Parkinson's disease [6–8]; chromium, nickel and copper cause DNA damage and epigenetic modifications associated with carcinogenesis [9,10]. More targeted effects have also been described: molybdenum affects the respiratory tract [11], cadmium disrupts steroidogenesis [10], and high cobalt reduces thyroid function [12].

These mechanisms result in multiple organ dysfunction [13, 14] and the development of chronic diseases [15]. Exposure to heavy metals cannot be evaluated in isolation from occupational, temporal and life-course factors. **Zhuoqi et al. (2025)**, using NHANES data, showed that higher urinary titanium was associated with poorer Mini-Mental State Examination scores in older adults after adjustment for potential confounders, illustrating how environmental exposures differentially affect more vulnerable individuals. It is hypothesized that the toxic effect of heavy metals on diverse organs can cause organ dysfunction and disease, which are known to be related to the development of frailty [1]. Large epidemiological studies have explored this connection, García-Esquinas found that blood lead levels a dose-respondent relationship with frailty in a sample of older adults [17]. Another study focused on middle age and older people found that urine levels of heavy metals was positively associated with frailty and furthermore found that those heavy metals were linked to a higher risk of mortality in frail middle-age and older people [18].

In the context of aging, social disadvantage amplifies the biological impact of environmental hazards, and frail older adults are particularly sensitive to these cumulative insults. Exposure to heavy metals is also related to social disadvantage, with higher levels of mercury, cadmium and lead seen in people with lower socioeconomic status [19].

The main objective of this work is to identify the differential impact of heavy-metal exposure on the frailty of Mexican older adults. In a pre-specified core analysis we focus on mercury, manganese, arsenic and lead — metals known to be concentrated near industrial sources and previously implicated in age-related deficits. In a comprehensive secondary analysis we additionally examine eight other metals measured in the same sub-sample (titanium, vanadium, chromium, cobalt, nickel, copper, molybdenum, silver and cadmium), and we test whether social vulnerability or sex modifies any observed association.

## Methods

### Design and sample

This is a secondary cross-sectional analysis of the Mexican Health and Aging Study (MHAS), a nationally representative cohort of adults aged 50 and older that began in 2001 and has completed seven waves of follow-up. In the 2018 wave, an ancillary sub-study measured heavy metals in hair samples obtained from 2,671 participants. In line with a previously reported MHAS protocol and to capture the cumulative life-course effect of these exposures, we did not restrict the analytic sample to individuals who were index participants; therefore, the age range of the analytic sample is 18 to 99 years. Participants missing the frailty index or the core covariates were excluded, yielding an analytic sample of 2,541 individuals (Fig. 1 of supplementary material).

### Heavy-metal measurements

Hair samples were prepared and analyzed by inductively coupled plasma–mass spectrometry (ICP-MS), following EPA method 6020B. A mass-spectrometer tuning solution containing representative elements (10 μg/L Ba, Bi, Ce, Co, In, Li, U) was used to verify instrument resolution and calibration. Quality control for each analytical batch included a method blank, a laboratory control sample, a spiked matrix and a duplicate. Three blank types were used: a calibration blank (acidified reage mmnt-grade water) to monitor drift, a method blank (matrix without analytes) processed in the same manner as samples, and a washing blank (nitric-acid solution) to flush the system. A 1000 μg/mL Hg standard and a Quality Control Standard 27 (QCS-27, 100 μg/mL; High Purity Standards) served as certified reference materials. Acceptance criteria were a recovery of ± 20% for the verification standard, ± 25% recovery and ≤ 20% relative percent difference for fortified samples, and blank concentrations below half of the quantification limit [20, 21]. A verification standard bracketed every ten samples. Only analyte values above the limit of detection were retained for analysis, and concentrations were natural-log transformed to reduce skewness before modelling.

Thirteen metals were quantified: mercury (Hg), titanium (Ti), vanadium (V), chromium (Cr), manganese (Mn), cobalt (Co), nickel (Ni), copper (Cu), arsenic (As), molybdenum (Mo), silver (Ag), cadmium (Cd) and lead (Pb). A composite index incorporating the four a priori metals (Hg, Mn, As, Pb) was additionally considered.

## Frailty index

A 60-item deficit-accumulation frailty index (FI-60) was constructed according to the standard procedure of Searle et al. (2008). Items cover multiple domains (comorbidity, activities of daily living, instrumental activities, self-rated health, sensory function, mental health, signs and symptoms) and are coded 0 (absence) to 1 (presence), partial scores allowed. The FI is the unweighted mean across available items; participants with more than 20% missing items were excluded. Observed FI values ranged from 0.05 to 0.68.

## Covariates

Covariates were chosen a priori based on their established relationship with frailty and heavy-metal exposure: sex (male/female), partnership status (with/without partner), years of formal schooling, self-reported regular physical exercise, occupational risk exposure (yes/no), smoking status (never, past, current), age in years, and the MHAS social vulnerability index (SVI) for the 2018 wave, a 54-item deficit-accumulation composite capturing social disadvantage.

### Statistical analysis

We first described the sample overall and by sex. Heavy-metal concentrations were summarized as mean (SD) and range on the log scale. Our primary analytic strategy treated the FI as a continuous outcome. For each of the 13 metals, we fitted (i) a crude linear regression (OLS) of the FI on the log-metal; (ii) an adjusted linear regression including all covariates; and (iii) a fractional-logit regression (generalized linear model with binomial family and logit link) to account for the bounded nature of the FI on [0, 1]. Heteroscedasticity-consistent (HC3) robust standard errors were used throughout. We additionally ran a single multivariable model that entered 12 metals simultaneously (arsenic was excluded because of the smaller complete-case sub-sample) to examine independent contributions.

We quantified variance inflation factors (VIF) to rule out collinearity, tested residual normality with Shapiro–Wilk and Anderson–Darling tests, assessed heteroscedasticity with the Breusch–Pagan test, and examined model specification with the Ramsey RESET test. Because we tested 13 metals, p-values were additionally corrected for false discovery rate (Benjamini–Hochberg). Effect modification was examined with multiplicative interaction terms between each log-metal and (a) the SVI and (b) sex; when a significant interaction was detected we computed simple slopes at the mean ± 1 SD of the moderator. Analyses were performed in Python 3.10 with statsmodels 0.14 and pandas 2.2; two-sided α = 0.05 was used for nominal significance.

#### Ethical issues

The MHAS protocol was approved by the Institutional Review Boards of the University of Texas Medical Branch and the Instituto Nacional de Estadística y Geografía. All participants provided written informed consent. The present secondary analysis used deidentified data and did not require additional ethics approval.

## Results

### Sample characteristics

Of the 2,671 participants with hair samples, 2,541 had complete data on the frailty index and core covariates. Mean age was 62.9 years (SD 11.1); 60.4% were women. The average frailty index was 0.24 (SD 0.11) and was higher in women (0.26) than in men (0.21). Educational attainment was modest (mean 6.7 years of schooling) and roughly one third reported regular exercise. Detailed sample characteristics by sex are shown in Table 1.

Valid hair concentrations were available for 642 to 2,565 participants depending on the metal (Table 1 of supplementary material). Concentrations were log-transformed before analysis. Pairwise correlations among log-metals were low to moderate (all |r| < 0.6), and variance inflation factors in the multivariable model were below 2 for every metal, indicating no collinearity problem.

## Heavy metals and the frailty index

In crude analyses, most metals showed an inverse association with the FI (Table 2). Mercury, vanadium, chromium, manganese, cobalt, nickel, copper, cadmium and lead were each significantly inversely associated with frailty at the nominal p < 0.05 level. After adjustment for sex, partnership, schooling, exercise, occupational risk, smoking status, age and SVI, these associations were markedly attenuated. Only cobalt retained a nominally significant inverse association with the FI (β = −0.006 per 1-log μg/g increase; 95% CI − 0.010 to − 0.001; p = 0.017). After false-discovery-rate correction across the 13 metals, no single metal reached statistical significance (p-FDR for cobalt = 0.22).

Results from the fractional-logit model were essentially identical to those of the OLS model, with average marginal effects matching OLS coefficients to within 0.0002 FI units, confirming robustness of the cobalt signal to model specification. The final adjusted model for cobalt is shown in Table 3. Besides cobalt, female sex, older age, fewer years of schooling, smoking, reduced occupational risk exposure and — most strikingly — higher social vulnerability were independently associated with a higher frailty index.

In the simultaneous multivariable model entering 12 metals together (n = 747 complete cases, R^2^ = 0.21), no individual metal reached statistical significance. Covariates (age, sex, schooling and SVI) carried the explanatory weight, consistent with substantial overlap in the information each metal provides.

## Effect modification by social vulnerability

Two metals showed evidence of interaction with the SVI: vanadium (β-interaction = − 0.061; 95% CI − 0.105 to − 0.016; p = 0.008) and copper (β-interaction = − 0.067; 95% CI − 0.114 to − 0.019; p = 0.007). After FDR correction across the 13 metals, both interactions were at the borderline of significance (pFDR ≈ 0.05). No other interaction was observed (Table 4).

### Effect modification by sex

No metal × sex interaction reached statistical significance. The nominally closest signals were for titanium (p = 0.092) and copper (p = 0.132), with a tendency for the coefficients to be more negative in women than in men; none survived correction for multiple testing. Importantly, the cobalt effect was similar in men (β = −0.007; p = 0.11) and women (β = −0.005; p = 0.07), indicating that the cobalt–frailty association identified in the full model does not depend on sex.

### Model diagnostics

Variance-inflation factors for all metals and covariates were below 2 in the multivariable model, ruling out collinearity. The Breusch–Pagan test indicated mild heteroscedasticity (p = 0.027 for the multivariable model; p < 0.001 for the adjusted model with cobalt), motivating the use of HC3 robust standard errors. Residuals were right-skewed (Shapiro–Wilk p < 10^−^^1^^0^), a pattern typical of bounded variables; the fractional-logit specification addressed this and produced virtually identical inferences. The Ramsey RESET test for mis-specification was non-significant (p = 0.11), suggesting no substantial functional-form misspecification.

## Discussion

In this large, population-based cross-sectional sample of Mexican adults, hair concentrations of thirteen heavy metals were broadly not associated with frailty once socio-demographic and behavioral confounders were taken into account. Of the four metals we had pre-specified as most relevant (mercury, manganese, arsenic and lead), all showed an inverse crude association with the FI that disappeared after adjustment. Only cobalt retained a nominally significant inverse association, which did not survive multiple-testing correction. Far more striking was the effect of social vulnerability: the SVI was the strongest single correlate of frailty in every model, with an effect roughly two orders of magnitude larger than that of any individual metal, and it shaped the size and direction of the trace-metal effects through interaction.

The absence of a robust positive association between the four a priori metals and frailty contrasts with the prior hypothesis that cumulative exposure to toxic metals would accelerate the deficit-accumulation process and previous evidence [17,18] Several interpretations should be considered. First, hair is a biomarker of medium-term exposure (approximately the preceding three to six months) and may fail to capture the relevant biological time window for slow-progressing conditions such as frailty. Second, our sample is community-dwelling and excludes institutionalized adults, which may exclude the most frail and the most highly exposed individuals. Third, if people with more advanced frailty reduce their food intake, their exposure through food sources might also drop — a form of reverse causation that would bias crude associations toward the inverse direction we observed. Fourth, most of the previous evidence comes from the same cohort, NHANES, hence the relationship could be limited to this population.

The cobalt signal, although not robust to multiple-testing correction, deserves mention. Cobalt is an essential component of vitamin B_12_, and lower hair cobalt might reflect poorer nutritional status rather than lower environmental exposure. Because nutritional deficiency is itself associated with greater frailty [23], this could generate a spurious inverse association or, alternatively, reveal a real protective effect of adequate cobalt intake. The observed effect size (~ 0.006 FI units per 1-log increase, or roughly 2.5% of the sample mean) is small and clinically modest; replication in longitudinal designs with serum B12 as a covariate is warranted.

The interaction between vanadium and copper with social vulnerability is one of the more suggestive findings. The inverse association of these two essential trace metals with frailty was effectively restricted to participants with high SVI; among those with low SVI the slope was flat or slightly positive. One interpretation is that the protective effect of adequate trace-metal availability becomes visible only in a context of broader deprivation, where essential-nutrient intake is lower and sensitivity to small changes is correspondingly higher. In better-off individuals, other determinants of frailty likely dominate and the trace-metal signal is drowned out. These findings are consistent with the broader literature on effect-measure modification by social context [24] and with emerging work on the vulnerability–exposure interaction in environmental epidemiology.

The lack of a metal × sex interaction is reassuring and suggests that the well-documented sex gap in frailty (women consistently score higher on the FI) does not operate through differential susceptibility to heavy metals in this sample. The sex effect is predominantly additive rather than multiplicative on the FI scale. This result is unexpected, since it conflicts with most of the literature [25, 26]. Sex directly related to occupational hazards, hence men and women have different types of exposure which should be reflected in heavy metals levels. One hypothesis is that these types of exposures are regionally bound, and so differ largely.

As far as we are aware, this is the first report that simultaneously examines thirteen heavy metals against a 60-item frailty index in a large Latin American cohort and formally tests effect modification by social vulnerability. Prior literature has typically focused on one or two metals at a time and has rarely probed interactions [16]. Strengths of our work include the sample size, the use of a deficit-accumulation FI which is biologically robust to the choice of individual items, the application of fractional-logit regression to respect the bounded nature of the outcome, and the formal correction for multiple testing.

Several limitations deserve explicit discussion. First, the cross-sectional design precludes causal inference; longitudinal analyses using future MHAS waves will be essential to disentangle temporality. Second, even though our sample is large, the number of participants with valid measurements of arsenic (n = 642), cadmium (n = 1,154) and molybdenum (n = 1,509) was smaller than for other metals, reducing power for those comparisons and explaining in part the modest fit of the simultaneous multivariable model. Multiple imputation under missing-at-random assumptions may be a productive extension. Third, we did not examine all metals with documented relevance to aging; rubidium, for example, has been linked to cognitive decline [16] but was not measured in MHAS. Fourth, we did not have biomarkers of kidney function (eGFR) or of nutritional status (B12, folate, zinc) which could act as both confounders and mediators of the cobalt and copper signals. Fifth, information on exposure to biomass smoke was not available and therefore could not be accounted for in the analyses. Because biomass exposure is a recognized source of environmental pollutants and may be associated with both metal exposure and health outcomes, residual confounding cannot be excluded. Sixth, classical multivariable regression may not be the optimal tool for analyzing correlated environmental mixtures; methods such as weighted quantile sum regression [27] quantile g-computation [28] and Bayesian kernel machine regression [29] would provide complementary evidence on joint effects, non-linearities and interactions that are difficult to capture in a standard GLM framework.

From a public-health perspective, our findings reinforce that the main modifiable determinants of frailty in Mexican older adults are social: years of schooling, smoking, exercise and — above all — the social vulnerability index. Environmental exposure to the metals examined here did not emerge as an independent driver of frailty in this sample, although the interaction findings suggest that such effects might materialize in the most disadvantaged subgroups and deserve targeted investigation. Future work should combine longitudinal follow-up, mixture-aware statistical methods and richer confounder control to determine whether cumulative heavy-metal exposure is a modifiable risk factor for frailty or merely a passive marker of broader social and nutritional disadvantage.

## Conclusions

In a large population-based sample of Mexican adults, individual hair concentrations of thirteen heavy metals were not robustly associated with the frailty index after comprehensive adjustment and multiple-testing correction. Cobalt showed a small inverse association that merits replication, and vanadium and copper had inverse effects that were confined to participants with high social vulnerability. Social disadvantage — not metal exposure — was the dominant correlate of frailty in every model we fitted. These results call for longitudinal, mixture-aware analyses and explicit evaluation of effect modification by social context in future environmental-aging research.

## Supplementary Material

Supplementary Files

This is a list of supplementary files associated with this preprint. Click to download.

- Supplementarymaterial.docx

## Figures and Tables

**Table 1:** Sample characteristics, overall and stratified by sex.

Variable	Total (N = 2,541)	Men (n = 1,007)	Women (n = 1,534)
Age, mean (SD), years	62.9 (11.1)	64.4 (10.5)	61.8 (11.1)
Has a partner, n (%)	1,828 (68.7)	843 (79.7)	985 (61.6)
Years of schooling, mean (SD)	6.7 (4.4)	7.3 (4.8)	6.3 (4.5)
Exercises, n (%)	951 (36.7)	500 (48.6)	451 (28.9)
Never smoked, n (%)	1,572 (62.4)	377 (37.4)	1,195 (77.9)
Past smoker, n (%)	669 (26.3)	433 (43.0)	236 (15.3)
Current smoker, n (%)	300 (11.8)	197 (19.5)	103 (6.7)
Binge drinking*, n (%)	845 (33.2)	335 (33.3)	510 (33.2)
Social vulnerability index (SVI), mean (SD)	0.44 (0.11)	0.43 (0.11)	0.44 (0.11)
Frailty index, mean (SD)	0.243 (0.11)	0.213 (0.10)	0.261 (0.11)

* Binge drinking defined as ≥ 2 drinks in one day for women and ≥ 3 drinks in one day for men.

SD = standard deviation; n = number.

**Table 2:** Crude and covariate-adjusted linear regression coefficients for each log-metal predicting the frailty index.

Metal	N	β crude	95% CI	p	β adj.	95% CI	p	N adj.
Hg	2237	−0.0062	−0.0097, −0.0026	0.001	0.0004	−0.0029, 0.0037	0.82	2194
Ti	2565	−0.0053	−0.0106, −0.0000	0.048	−0.0030	−0.0077, 0.0018	0.22	2516
V	2056	−0.0090	−0.0143, −0.0038	0.001	−0.0031	−0.0079, 0.0017	0.20	2014
Cr	2426	−0.0135	−0.0182, −0.0087	< 0.001	0.0002	−0.0048, 0.0052	0.93	2377
Mn	2505	−0.0059	−0.0097, −0.0021	0.002	−0.0024	−0.0059, 0.0011	0.19	2456
Co	1455	−0.0113	−0.0167, −0.0059	< 0.001	−0.0056	−0.0103, −0.0010	0.02*	1428
Ni	2482	−0.0097	−0.0141, −0.0054	< 0.001	−0.0019	−0.0060, 0.0021	0.34	2433
Cu	2564	−0.0075	−0.0130, −0.0020	0.008	−0.0034	−0.0084, 0.0017	0.19	2515
As	642	−0.0059	−0.0160, 0.0042	0.250	0.0052	−0.0045, 0.0149	0.29	629
Mo	1509	−0.0049	−0.0114, 0.0016	0.140	0.0040	−0.0022, 0.0101	0.21	1477
Ag	1771	−0.0029	−0.0063, 0.0005	0.100	−0.0007	−0.0039, 0.0024	0.64	1736
Cd	1154	−0.0089	−0.0147, −0.0031	0.003	−0.0028	−0.0082, 0.0027	0.32	1126
Pb	2545	−0.0082	−0.0119, −0.0046	< 0.001	−0.0021	−0.0055, 0.0014	0.23	2496

P = unstandardized regression coefficient expressed as the change in the frailty index (0–1 scale) per one-unit increase in log(metal). Crude models include only the metal; adjusted models additionally control for sex, partnership, years of schooling, exercise, occupational risk, smoking status (factor), age and the social vulnerability index. Standard errors are heteroscedasticity-consistent (HC3). Values in bold red are statistically significant at p < 0.05 (nominal).

*** p < 0.001;

** p < 0.01;

* p < 0.05

**Table 3:** Final adjusted fractional-logit model for log(cobalt) and the frailty index (N = 1,428).

Predictor	β (logit scale)	95% CI	p-value
log(Co)	−0.030	−0.056, −0.005	0.021*
Female (ref: male)	0.311	0.235, 0.387	<0.001***
Has a partner	0.028	−0.039, 0.095	0.410
Years of schooling	−0.016	−0.024, −0.009	<0.001***
Exercises regularly	0.097	0.034, 0.161	0.003**
Occupational risk	−0.067	−0.125, −0.009	0.024*
Past smoker (ref: never)	0.078	0.003, 0.152	0.041*
Current smoker	0.054	−0.038, 0.146	0.252
Age (years)	0.009	0.006, 0.012	< 0.001***
Social vulnerability index (SVI)	1.413	1.105, 1.722	< 0.001***

Model: generalized linear model with binomial family and logit link, HC3 robust standard errors. McFadden pseudo-R^2^ = 0.02; OLS R^2^ from the equivalent linear specification = 0.19. Coefficients are on the logit scale; the sign and significance of the average marginal effects on the FI 0–1 scale match the β's above.

*** p < 0.001;

** p < 0.01;

* p < 0.05

**Table 4:** Interaction between each log-metal and the social vulnerability index for predicting the frailty index.

Metal	β interaction	95% CI	p-value
Hg	0.007	−0.022, 0.036	0.640
Ti	−0.042	−0.090, 0.006	0.084
V	−0.061	−0.105, −0.016	0.008**
Cr	−0.014	−0.050, 0.022	0.450
Mn	−0.028	−0.060, 0.005	0.094
Co	−0.003	−0.043, 0.037	0.880
Ni	−0.017	−0.052, 0.018	0.340
Cu	−0.067	−0.114, −0.019	0.007**
As	0.036	−0.049, 0.122	0.400
Mo	0.010	−0.042, 0.062	0.710
Ag	−0.008	−0.038, 0.022	0.600
Cd	0.004	−0.040, 0.049	0.850
Pb	−0.001	−0.036, 0.034	0.950

Probing the two significant interactions at low (mean - 1 SD), medium (mean) and high (mean + 1 SD) SVI revealed that the inverse association of vanadium and copper with the FI was confined to participants with high social vulnerability (Table 6). At low SVI both slopes were slightly positive and non-significant. At high SVI, a 1-log increase in vanadium was associated with a 0.009-unit decrease in FI (p = 0.010), and a 1-log increase in copper was associated with a 0.012-unit decrease in FI (p = 0.007).

*** p < 0.001;

** p < 0.01;

* p < 0.05

**Table 5:** Simple slopes of vanadium and copper on the frailty index at low, medium and high levels of the social vulnerability index.

Metal	SVI level	β	95% CI	p
Vanadium (V)	Low SVI (mean - 1 SD, 0.33)	0.0040	−0.003, 0.010	0.260
	Medium SVI (mean, 0.44)	−0.0030	−0.008, 0.002	0.260
	High SVI (mean + 1 SD, 0.55)	−0.0090	−0.017, −0.002	0.010*
Copper (Cu)	Low SVI (mean - 1 SD, 0.33)	0.0030	−0.003, 0.008	0.400
	Medium SVI (mean, 0.44)	−0.0050	−0.010, 0.000	0.070
	High SVI (mean + 1 SD, 0.55)	−0.0120	−0.021, −0.003	0.007**

*** p < 0.001;

** p < 0.01;

* p < 0.05

## Data Availability

The data that support the findings of this study are available from the Mexican Health and Aging Study (MHAS) repository (https://www.mhasweb.org/). Data are publicly available upon registration and compliance with the data use agreement. The authors did not generate new data for this study.
